# Metabolic Activity of Radish Sprouts Derived Isothiocyanates in *Drosophila melanogaster*

**DOI:** 10.3390/ijms17020251

**Published:** 2016-02-18

**Authors:** Nieves Baenas, Stefanie Piegholdt, Anke Schloesser, Diego A. Moreno, Cristina García-Viguera, Gerald Rimbach, Anika E. Wagner

**Affiliations:** 1 Institute of Human Nutrition and Food Science, University of Kiel, Hermann-Rodewald-Strasse 6, 24118 Kiel, Germany; nbaenas@cebas.csic.es (N.B.); piegholdt@foodsci.uni-kiel.de (S.P.); schloesser@foodsci.uni-kiel.de (A.S.); rimbach@foodsci.uni-kiel.de (G.R.); 2 Department of Food Science and Technology, CEBAS-CSIC, Campus de Espinardo 25, 30100 Murcia, Spain; dmoreno@cebas.csic.es (D.A.M.); cgviguera@cebas.csic.es (C.G.-V.)

**Keywords:** *Brassicaceae*, sulforaphene, radish, *spargel*, energy metabolism

## Abstract

We used *Drosophila melanogaster* as a model system to study the absorption, metabolism and potential health benefits of plant bioactives derived from radish sprouts (*Raphanus sativus* cv. Rambo), a *Brassicaceae* species rich in glucosinolates and other phytochemicals. Flies were subjected to a diet supplemented with lyophilized radish sprouts (10.6 g/L) for 10 days, containing high amounts of glucoraphenin and glucoraphasatin, which can be hydrolyzed by myrosinase to the isothiocyanates sulforaphene and raphasatin, respectively. We demonstrate that *Drosophila melanogaster* takes up and metabolizes isothiocyanates from radish sprouts through the detection of the metabolite sulforaphane-cysteine in fly homogenates. Moreover, we report a decrease in the glucose content of flies, an upregulation of *spargel* expression, the *Drosophila* homolog of the mammalian PPARγ-coactivator 1 α, as well as the inhibition of α-amylase and α-glucosidase *in vitro*. Overall, we show that the consumption of radish sprouts affects energy metabolism in *Drosophila melanogaster* which is reflected by lower glucose levels and an increased expression of *spargel*, a central player in mitochondrial biogenesis. These processes are often affected in chronic diseases associated with aging, including type II diabetes mellitus.

## 1. Introduction

**Figure 1:**
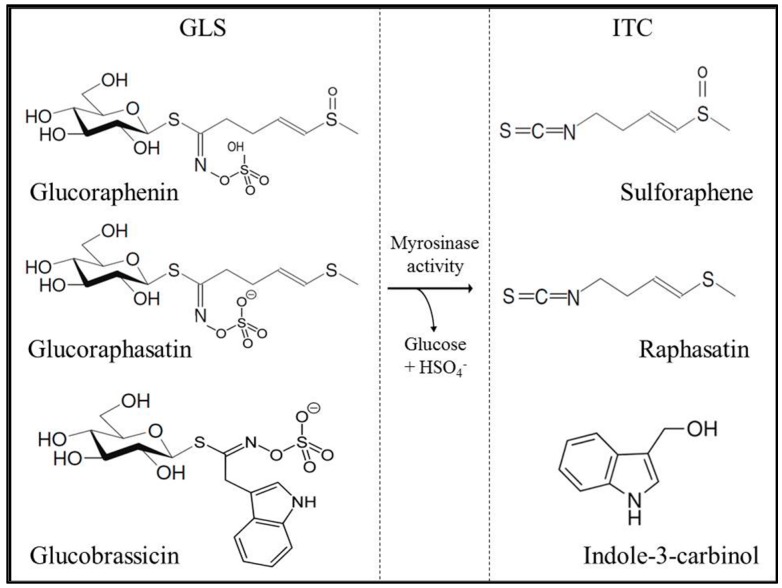
Glucosinolates in radish sprouts and their corresponding hydrolysis to isothiocyanates.

Obesity and related diseases, such as diabetes and cardiovascular diseases, are a growing and serious health problem in both industrialized and developing countries [1]. The consumption of cruciferous plants (*Brassicaceae* family) has been associated with beneficial metabolic effects, although the underlying cellular and molecular mechanisms have not yet been fully elucidated [2,3,4]. *Brassicaceae* contain high amounts of glucosinolates (GLS), bioactive compounds that are enzymatically hydrolyzed to several breakdown products, including isothiocyanates (ITC, Figure 1). Treatment with indole-3-carbinol (I3C) and 3,3′-diindolylmethane (DIM), hydrolysis products of the GLS glucobrassicin, has been shown to significantly decrease blood glucose levels in C57BL/6 mice receiving a high fat diet [5]. Furthermore, patients suffering from type II diabetes exhibited significantly improved fasting glucose and lower insulin levels as well as an augmented homeostasis model assessment of insulin resistance (HOMA-IR) index following the consumption of 10 g/day broccoli sprout powder for four weeks [6]. Radish sprouts have been widely studied because of their high content of potentially health-promoting GLS [7,8]. The main compounds glucoraphenin and glucoraphasatin, belonging to the aliphatic group of GLS, are hydrolyzed by the plant endogenous enzyme myrosinase (thioglucoside glucohydrolase, EC 3.2.1.147), following plant tissue disruption to sulforaphene (SFE; 4-methylsulfinyl-3-butenyl ITC) and raphasatin (RPS; 4-methylsulfanyl-3-butenyl ITC), respectively [9]. Also, indole GLS, such as glucobrassicin, 4-hydroxyglucobrassicin and 4-methoxyglucobrassicin, are present in radish sprouts and have been studied because of their breakdown product I3C, which has been associated with improved glucose tolerance and modulated expression of adipokines and lipogenic-associated gene products, including acetyl-CoA carboxylase and peroxisome proliferator-activated receptor-γ [10].

In mammals ITC are known to be metabolized in the enterocytes and the liver through the mercapturic acid pathway. Initially, a reaction between the –N=C=S group of the ITC and the cysteine sulfhydryl group of glutathione (GSH) catalyzed by glutathione-S-transferase (GST) takes place. Next, hepatic enzyme modifications of the GSH moiety to cysteinylglycine (–cys–gly), cysteine (cys), and *N*-acetyl-cysteine (NAC) conjugates are formed in the kidney of mammalian species [11]. Little is known about the absorption, metabolism and metabolic effects of ITC in model organisms. In this study, the fruit fly *Drosophila melanogaster* is used as a model system for studying the absorption and metabolism of ITC in lyophilized radish sprouts. In addition, the bioactivity of these compounds in terms of glucose and energy metabolism is evaluated.

## 2. Results and Discussion

### 2.1. Glucosinolate and Isothiocyanate Content in Radish Sprouts

The GLS profile of *Brassicaceae* species varies by genotype [12]. Radish sprouts (*Raphanus sativus* cv. Rambo) display a characteristic GLS profile, which has been extensively studied because of its hydrolysis products, ITC and indoles. ITC and indoles have been suggested to have protective effects on disease development through anti-inflammatory, chemopreventive and epigenetic pathways [13]. The concentrations of GLS are the highest in seeds and decline exponentially with sprout development. Aliphatic GLS are the predominant compounds in eight-day-old radish sprouts. Glucoraphenin and glucoraphasatin account for approximately 40% and 50% of the total GLS, respectively, as summarized in Table 1.

Following endogenous myrosinase hydrolysis of glucoraphenin and glucoraphasatin, these compounds released SFE and RPS, respectively. RPS is the oxidized counerpart of SFE and was not detected with our UHPLC method because a commercial standard was not available. However, this compound has been reported to be highly unstable, with a half-life of less than 30 min [9]. Besides its instability RPS has been reported to significantly induce the expression of detoxifying enzmyes [14]. In addition to SFE, we also detected sulforaphane (SFN) in the hydrolyzed samples. The detection of SFN is interesting because glucoraphanin, the precursor of SFN, was not detected in our radish sprouts. However, these molecules only differ chemically by one double bond, suggesting that SFN exists as a natural product or that the presence of SFN is an artifact of the analytical technique, as we did not find any reports of this conversion taking place. We also detected indole GLS, which accounts for 15% of the total GLS in our radish sprouts. In particular, 4-hydroxyglucobrassicin and 4-methoxyglucobrassicin were present at higher amounts than glucobrassicin and neoglucobrassicin (Table 1). Their main hydrolysis compound, I3C (1 mg/100 g F.W. (Fresh Weight)), has been shown to lead to decreases in body weight via its effect on fat accumulation and blood glucose levels in mice [5,10].

### 2.2. Evaluation of Food Intake and Fitness in Drosophila melanogaster

**Figure 2:**
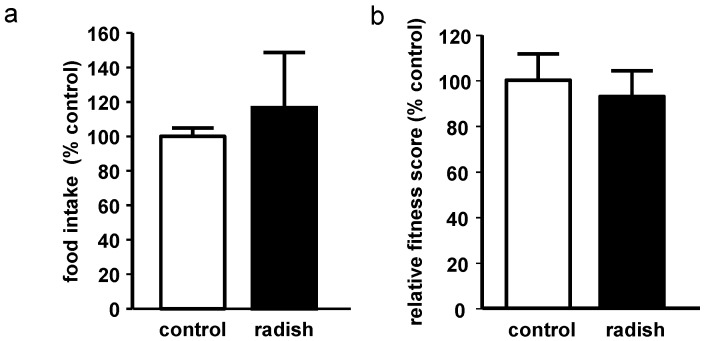
Effect of 10-day radish sprout supplementation on male *Drosophila melanogaster*. (**a**) relative food intake analyzed by the gustatory assay (*n* = 3 + SEM; extraction from 3 × 15 flies); (**b**) relative fitness score detected by the RING assay (*n* = 3 + SEM).

Food intake did not differ between control flies and radish sprout-treated flies (Figure 2a). Climbing ability, as a marker of overall fitness of the flies, did not show statistically significant differences between groups (Figure 2b), indicating that supplementation of the SY medium with lyophilized radish sprouts did not affect the movement of the flies.

### 2.3. Sulforaphene, Sulforaphane, Indole-3-Carbinole, and Sulforaphane-Cysteine Concentrations in Fly Homgenates

The isothiocyanates SFE, SFN and I3C were present in our flies in concentrations of nanomol per gram on a fresh weight basis. Thus, under the conditions investigated, the natural conversion of glucosinolates to isothiocyanates, by the plant-derived enzyme myrosinase but also by gut microflora-derived myrosinase, may have occurred [3].

**Figure 3:**
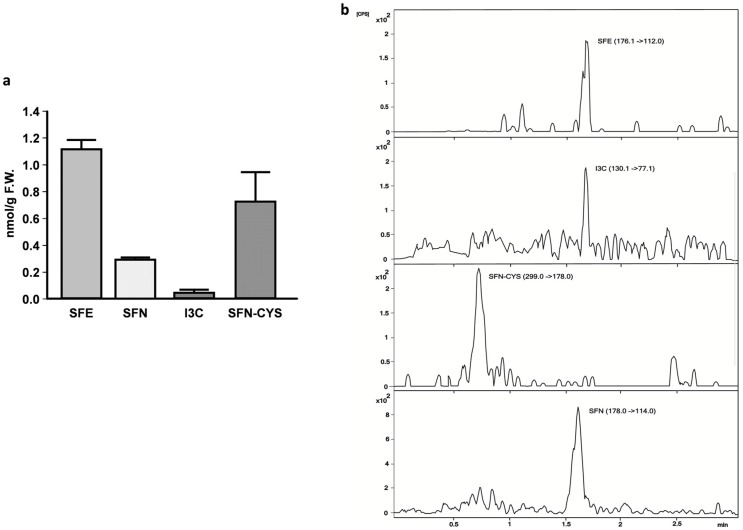
(**a**) Metabolites present in *Drosophila melanogaster* following the consumption of radish sprouts for 10 days; (**b**) Representative chromatogram of metabolites found in *Drosophila melanogaster* following the consumption of radish sprouts for 10 days. F.W. = fresh weight, SFE = sulforaphene, SFN = sulforaphane, I3C = indole-3-carbinole, SFN–CYS = sulforaphane–cysteine; *n* = 3 + SD.

Flies principally accumulate the ITC SFE (1.11 nmol/g F.W. in flies, Figure 3). SFN was also found to be present in radish sprout-fed flies, but we cannot confirm whether SFN derives from the radish sprout extract, whether it is formed in the organism or whether SFN is formed due to a spontaneous conversion between SFE and SFN. Support for a metabolic origin of SFN can be obtained by finding an additional SFN conjugate—SFN–cysteine (SFN–CYS)—which was also detectable in our flies (0.7 nmol/g F.W.). This finding suggests that GLS and ITC were metabolized in the flies and that the initial reaction between ITC and GSH may be performed as a first step in SFN–CYS conjugation [11]. It has been shown that SFN treatment elevated cellular GSH levels [15,16] which prevents the accumulation of free radicals inside the cells and thereby reduces oxidative stress, which is generally associated with the development and progression of diabetes and its complications [17]. Interestingly, the distribution of GLS and ITC present in radish sprouts is partly reflected in ITC levels of our flies. The presence of the SFN–CYS metabolite in radish sprouts fed flies suggests that the metabolization of brassica-derived bioactive compounds in *Drosophila melanogaster* is similar to mammalian species.

### 2.4. Inhibition of α-Amylase and α-Glucosidase in Vitro by Radish Sprouts

An aqueous extract of lyophilized radish sprouts had an inhibitory effect on α-amylase and α-glucosidase activity *in vitro* (Table 2). The calculated concentration of the extract required to achieve half of the maximal inhibitory concentration (IC_50_) was higher in the α-glucosidase inhibition assay (60.7 ± 1.2 mg/mL) than in the α-amylase inhibition assay (33.8 ± 4.0 mg/mL). The concentration of 10.6 mg/mL radish sprouts which we have used in our *Drosophila melanogaster* experiments resulted in a 23% inhibition of the α-amylase activity *in vitro*, while the *in vitro* α-glucosidase activity was not affected (data not shown).

Inhibition of the enzyme α-amylase in the intestines delays the degradation of starch and oligosaccharides to monosaccharides before they can be absorbed. The enzyme α-glucosidase catalyzes the final step in the digestion and breakdown of carbohydrates; thus, its inhibition can be effective for the regulation of Type II diabetes through the control of glucose absorption [18]. The inhibition of α-glucosidase may retard the digestion and absorption of carbohydrates. In addition, it may suppress post-prandial hyperglycemia, decrease calorie uptake, and result in lower levels of glucose in *Drosophila melanogaster*. Thus, radish sprouts may improve glucose homeostasis and provide a dietary strategy to control hyperglycemia in diabetic and obese patients. However, additional evaluation of the *in vivo* potential of anti-diabetic activity of radish sprouts bioactives is necessary to verify these beneficial effects.

### 2.5. Energy Metabolism in Drosophila melanogaster

**Figure 4:**
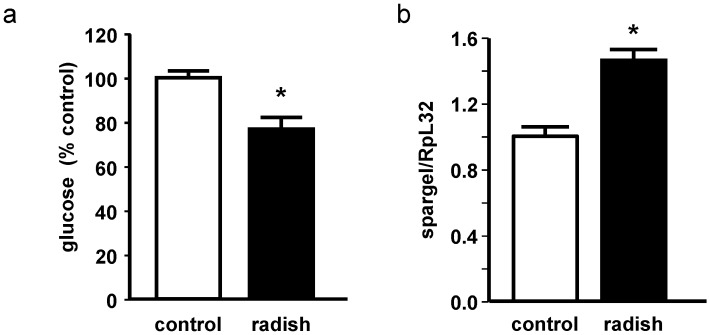
Effect of 10-day radish sprout supplementation on male *Drosophila melanogaster*. (**a**) relative glucose levels (*n* = 9 + SEM; extraction from 9 × 5 flies); (**b**) relative mRNA levels of *spargel* related to the housekeeping gene *RpL32* (*n* = 3 + SEM; extraction from 3 × 5 flies). * indicates significant differences between control and radish sprout-fed flies (*p* < 0.05, Student’s *t*-test).

The glucose levels in our radish sprout-treated flies were significantly lower than those in control flies (Figure 4a). This finding is consistent with results obtained by Okulicz and co-workers [19], who observed that I3C, a breakdown product also present in radish sprouts, affects glucose uptake in adipocytes under basal as well as under insulin-stimulated conditions.

Because we have detected inhibition of α-amylase *in vitro* by radish sprouts and decreased glucose levels in our flies, we suggest that intestinal glucose absorption decreased as a consequence of radish sprout treatment. Glucose has been described to be a pro-aging factor and to interact with several age-associated processes in the organism [20,21]. High glucose availability has been shown to shorten life span whereas a glucose restriction increased life span in the model organism *Caenorhabditis elegans* [22]. Interestingly, PGC-1α has been suggested to be involved in the regulation of glucose homeostasis in mammals [23,24]. Along with decreased glucose levels, we also showed that there was an upregulation of the PGC-1α homologous gene *srl* in *Drosophila* (Figure 4b). PGC-1α/*srl* plays an important role in the stimulation of mitochondrial biogenesis, in the reduction of ROS levels in enterocytes and stem cells, in the induction of several ROS-detoxifying enzymes and in the maintenance of optimal intestinal homeostasis [25]. Furthermore, an overexpression of PGC-1α/*srl* in the intestine of *Drosophila melanogaster* has been reported to be associated with life span extension [25].

Thus, we could not detect an improved resistance of our radish sprout-treated flies against both hydrogen peroxide and paraquat-induced stress (data not shown), which may be attributed to the relatively short intervention period of 10 days. However, there appears to be a connection between insulin resistance and mitochondrial dysfunction because patients suffering from type II diabetes have been reported to exhibit lower levels of mitochondria-related OXPHOS genes and PGC-1α in their skeletal muscles [26,27]. In addition, insulin has been shown to induce PGC-1α expression in skeletal muscle [28]. Therefore, insulin resistance may lead to a decrease in PGC-1α expression and mitochondrial dysfunction, which, in turn, increases insulin resistance further [26]. An increase in PGC-1α expression may provide a means to disrupt this vicious circle so that glucose homeostasis in diabetic and obese patients can be improved. Furthermore, Fernandes and co-workers have demonstrated that there is an approximately 40% increase in cellular PGC-1α levels as a result of SFN administration in cultured rat cardiac myoblasts [29]. Thus, we suggest that radish sprout-derived bioactives may affect glucose homeostasis—at least partially—through the modulation of PGC-1α-expression.

## 3. Materials and Methods

### 3.1. Radish Sprout Production

Red radish sprouts (*Raphanus sativus* cv. Rambo) were germinated for 8 days, according to the protocol of Baenas *et al.* [7]. Briefly, sprouts were collected, flash frozen in liquid nitrogen, and stored at −80 °C prior to analyses. Samples were then lyophilized and ground into a fine powder before being extracted for analyses and used as fly food supplement.

### 3.2. Analyses of GLS and ITC in Radish Sprouts and Drosophila Melanogaster by HPLC-DAD-ESI-MSn and UHPLC-QqQ-MS/MS

GLS in radish sprouts were extracted and quantified by HPLC-DAD-ESI-MSn, according to the protocol of Baenas [7]. Briefly, GLS were first identified following their MS2 [M–H]-fragmentations and were then quantified following their UV spectra and order of elution as previously described for the acquisition conditions. Sinigrin and glucobrassicin were used as external standards for aliphatic and indole GLS, respectively. ITC in radish sprouts were extracted according to the protocol of Cramer and Jeffery [30] and quantified by UHPLC-QqQ-MS/MS, according to the protocol of Dominguez-Perles *et al.* [31]. Also, this method was used to analyze metabolites in *Drosophila melanogaster*. First, 200 mg of flies were extracted with 5 mL MeOH:H_2_O (70:30), mashed with a mortar until a homogenous liquid was obtained, filtered by 0.22 µm PVDF and analyzed with UHPLC-QqQ-MS/MS. The standards SFE, SFN, SFN–CYS and I3C were identified and quantified using MRM transitions and positive or negative ESI mode for quantification and confirmation of the target analytes. Analyses of GLS and ITC of radish sprouts were conducted in triplicate.

### 3.3. In Vitro α-Amylase and α-Glucosidase Assay of Radish Sprouts

The α-amylase and α-glucosidase inhibition assay was performed using a protocol modified from Phan *et al.* [32]. Samples of lyophilized radish sprouts (*n* = 3) were extracted with distilled water. The extracts were tested for α-glucosidase and α-amylase inhibition as previously described [33]. Acarbose was used as a positive control and was equally dissolved in distilled water. IC_50_ values were calculated using the program GraphPad prism (La Jolla, CA, USA).

### 3.4. Drosophila melanogaster Stocks and Treatment

In the present study, W^1118^ *Drosophila melanogaster* was used for the experiments. Flies were maintained under conventional conditions on sugar yeast medium (SY) containing 10% sucrose (Carl Roth, Karlsruhe, Germany), 10% inactive dry yeast, 2% agar, 0.3% nipagin (all Dominique Dutscher SAS, Brumath, France), and 0.3% propionic acid (Carl Roth) in a climate chamber (HPP 1018, Memmert, Schwabach, Germany) under the following constant conditions: a temperature of 25 °C, relative humidity of 60% and 12-h day/night cycle. For all of the experiments, age-matched flies from synchronized eggs were used [34]. The SY medium was supplemented with radish sprouts at a concentration of 10.6 g/L, containing 50 µmol/L of the ITC SFE.

### 3.5. Gustatory Assay

This method was performed to exclude differences in food intake between control flies and radish sprouts treated flies. The gustatory assay was performed as described earlier [34]. In brief, 15 flies were kept on SY medium or SY+radish sprouts. Both were supplemented with 0.2% *w*/*v* sulforhodamine B sodium salt (Sigma-Aldrich, Steinheim, Germany) and kept under standard conditions for 500 min. Next, the flies were homogenized in PBS (Life Technologies by Thermo Fisher Scientific, Darmstadt, Germany) plus 1% Triton™ Χ-100 (Sigma-Aldrich) using a Qiagen TissueLyser II (Hilden, Germany). The flies were then centrifuged, and the absorbance was measured in an Infinite 200 spectrophotometer (Tecan, Crailsheim, Germany) at 535/25 nm excitation and 590/20 nm emission wavelength.

### 3.6. Negative Geotaxis Assay: Climbing Activity

Climbing ability was considered to be an indicator of overall fitness of the flies, which were maintained under control conditions (SY) and under SY+radish sprouts for 10 days. On day 10, flies were transferred into empty vials to perform the rapid iterative negative geotaxis (RING)-assay as previously described [33].

### 3.7. Glucose Analysis

Flies were maintained either on SY (*n* = 20) or SY+radish sprouts under standard conditions for 10 days. Five flies per sample were homogenized in PBS/1% Triton™ X-100 using a Qiagen TissueLyser II. The supernatant was removed and analyzed for glucose content with Fluitest^®^GLU (Analyticon Biotechnologies, Lichtenfels, Germany) according to the manufacturer’s protocol. Sample concentrations were calculated via the standard curve and related to the corresponding fly weights. The weighing of flies was performed using previously described methods [35].

### 3.8. Real-Time PCR

Total RNA was isolated from 5 flies per sample using peqGOLD TriFast™ (Peqlab, Erlangen, Germany) according to the manufacturer’s protocol. RNA concentration was determined using a NanoDrop^®^ spectrophotometer (Thermo Scientific, Schwerte, Germany). Primer sequences for *Drosophila melanogaster spargel* (*srl*) are described elsewhere [36]. Primers for the housekeeping gene *Drosophila melanogaster Ribosomal protein L32* (*RpL32*) (forward 5′-GGCAAGCTTCAAGATGACCA-3′; reverse 5′-GTTCGATCCGTAACCGATGT-3′) were designed by Primer3 software (Whitehead Institute for Biomedical Research, Cambridge, MA, USA). All primers were purchased from MWG Biotech (Ebersberg, Germany). Real-time PCR was performed using the SensiFastTM SYBR^®^ No-ROX One-Step kit (Bioline, Luckenwalde, Germany) on a Rotor-Gene 6000 cycler (Corbett Life Science, Sydney, Australia). The expression of *srl* was related to the expression of the housekeeping gene *RpL32*.

### 3.9. Statistics

The results are presented as the mean ± SEM unless otherwise indicated. All data were analyzed using SPSS software (Statistical Package for the Social Sciences, IBM, Armonk, NY, USA). The significance of the differences between the control and radish sprout-treated flies was evaluated using a Student’s *t*-test. All data were tested for normality of distribution (Shapiro–Wilk) and the homogenicity of variances (Levene). Significance was accepted at *p*-values <0.05.

## 4. Conclusions

In the present study, we showed, for the first time, that plant bioactives present in radish sprouts are absorbed and hydrolyzed by *Drosophila melanogaster* either due to the presence of plant-derived or *Drosophila*-derived microbial myrosinase in the gut. The presence of SFN–CYS in fly homogenates suggests that ITC are metabolized by fruit flies. The intake of radish sprouts decreased the glucose content in our flies and increased *srl* expression levels, thereby modulating energy metabolism. Understanding the factors that determine the absorption and effects of ITC in *in vivo* models is critical for identifying effective dosages that can be used in nutritional studies. As we have only analyzed flies on a radish sprout-supplemented diet for 10 days, analyses of flies receiving radish sprout-supplemented food for a longer period are sorely needed to assess the robustness of our results. To confirm the involvement of *srl* in the potential beneficial metabolic effects of radish sprouts, life span studies are important. Additionally, studies in mammalian species are needed to confirm our results that were obtained in the model organism *Drosophila melanogaster*.

## Figures and Tables

**Table 1:** Quantification of glucosinolates and isothiocyanates in radish sprouts.

Glucosinolate Content in Radish Sprouts (mg/100 g F.W.)	
Glucoraphenin	202 ± 18.3
4-Hydroxyglucobrassicin	19.9 ± 1.32
Glucoerucin	8.74 ± 1.85
Glucoraphasatin	250 ± 23.5
Glucobrassicin	6.48 ± 0.36
4-Methioxyglucobrassicin	19.5 ± 0.72
Neoglucobrassicin	6.51 ± 0.31
Aliphatic GLS	461 ± 42.3
Indole GLS	52.4 ± 1.97
Total	514 ± 44.0
**Isothiocyanate Content in Radish Sprouts (mg/100 g F.W.)**	
Sulforaphene	9.93 ± 0.01
Sulforaphane	0.97 ± 0.02
Indole-3-carbinol	1.00 ± 0.09
Total	11.9 ± 0.11

Mean values (*n* = 3) ± SD. F.W. (Fresh Weight).

**Table 2:** *In vitro* α-glucosidase and α-amylase inhibitory activity of an aqueous extract of radish sprouts.

Inhibitory Activity of Radish Sprouts	
α-Glucosidase IC_50_	60.8 ± 1.16 (mg/mL)
α -Amylase IC_50_	33.8 ± 4.00 (mg/mL)

Mean values (*n* = 3) ± SD; IC_50_ (concentration which shows 50% of the inhibitor’s response).

## Abbreviations

GLS: glucosinolates
ITC: isothiocyanates
PGC1α: PPARγ co-activator 1 α
PPARγ: peroxisome proliferator activated receptor γ
GSH: glutathione
GST: glutathione-S-transferase
NAC: *N*-acetyl-cysteine
cys: cysteine
cys–gly: cysteinylglycine
SFE: sulforaphene
SFN: sulforaphane
RPS: raphasatin
SFN–CYS: sulforaphane-cysteine
*RpL*32: *Drosophila melanogaster* ribosomal protein L32
*Srl*: spargel
